# Personalized Stress Detection Using Biosignals from Wearables: A Scoping Review

**DOI:** 10.3390/s24103221

**Published:** 2024-05-18

**Authors:** Marco Bolpagni, Susanna Pardini, Marco Dianti, Silvia Gabrielli

**Affiliations:** 1 Human Inspired Technology Research Centre, University of Padua, 35121 Padua, Italy; 2 Digital Health Research, Centre for Digital Health and Wellbeing, Fondazione Bruno Kessler, 38123 Trento, Italy; spardini@fbk.eu (S.P.); dianti@fbk.eu (M.D.); sgabrielli@fbk.eu (S.G.)

**Keywords:** personalized stress detection, stress, wearables, Internet of Things (IoT), artificial intelligence (AI), scoping review, PRISMA framework

## Abstract

Stress is a natural yet potentially harmful aspect of human life, necessitating effective management, particularly during overwhelming experiences. This paper presents a scoping review of personalized stress detection models using wearable technology. Employing the PRISMA-ScR framework for rigorous methodological structuring, we systematically analyzed literature from key databases including Scopus, IEEE Xplore, and PubMed. Our focus was on biosignals, AI methodologies, datasets, wearable devices, and real-world implementation challenges. The review presents an overview of stress and its biological mechanisms, details the methodology for the literature search, and synthesizes the findings. It shows that biosignals, especially EDA and PPG, are frequently utilized for stress detection and demonstrate potential reliability in multimodal settings. Evidence for a trend towards deep learning models was found, although the limited comparison with traditional methods calls for further research. Concerns arise regarding the representativeness of datasets and practical challenges in deploying wearable technologies, which include issues related to data quality and privacy. Future research should aim to develop comprehensive datasets and explore AI techniques that are not only accurate but also computationally efficient and user-centric, thereby closing the gap between theoretical models and practical applications to improve the effectiveness of stress detection systems in real scenarios.

## 1. Introduction

Stress can be described as a state of unease or mental strain that arises from challenging circumstances. It is a natural human reaction that urges us to confront difficulties and threats in our lives. Experiencing stress is part of everyone’s life; nonetheless, how we manage stress significantly influences our overall well-being [1]. According to the American Psychological Association (APA) [2], stress is a typical response to the demands of daily life. However, it can become detrimental when it disrupts our day-to-day activities. In such instances, stress can make it challenging to unwind and can be accompanied by a spectrum of emotions, such as anxiety and irritability. Concentration may become difficult when under stress, and physical symptoms like headaches, body aches, upset stomach, or sleep disturbances may manifest. Appetite changes, either a loss of appetite or increased eating, can also be observed [3]. Prolonged stress can exacerbate existing health issues and may lead to heightened use of substances like alcohol, tobacco, and others [4]. Stressful situations can also trigger or worsen mental health conditions, particularly anxiety and depression, necessitating access to healthcare [5]. From a biological point of view, stress is a complex physiological response to a perceived threat or challenge. This response is part of the body’s natural adaptive mechanism, often referred to as the “fight-or-flight” response, which has evolved to help individuals cope with potentially dangerous situations [6]. The primary biological player in the stress response is the endocrine system, particularly the release of stress hormones, such as cortisol and adrenaline [7]. When an individual perceives a situation as stressful, the brain’s amygdala interprets this as a potential threat, and the hypothalamus–pituitary–adrenal (HPA) axis is activated. The adrenal glands release stress hormones, primarily cortisol and adrenaline, into the bloodstream, which trigger a cascade of physiological responses throughout the body, including increased heart rate and blood pressure, dilation of the airways, diversion of blood flow away from non-essential functions, release of glucose into the bloodstream, heightened alertness, and increased perception of pain [8]. The stress response prepares the body to either confront the perceived threat (fight) or escape from it (flight) by mobilizing energy reserves for a rapid and robust response. Once the perceived threat is resolved, the body initiates mechanisms to halt the stress response, cortisol levels drop, and the body returns to a state of equilibrium (homeostasis). Although clinical practice guidelines have not yet established a definitive standard for assessing stress through biofeedback, they highlight the importance of integrating multiple biosignals (Electroencephalogram (EEG), Electrodermal Activity (EDA), and Heart Rate Variability (HRV)) with traditional psychological assessment tools, such as standardized questionnaires. These assessments should be customized to individual needs and calibrated to each subject based on baseline measurements [9,10]. Similarly, researchers utilize a variety of methods to measure stress responses, including self-reports, behavioral cues, and physiological changes induced by stress. Self-report tools like the Perceived Stress Scale (PSS) [11] assess the emotional burden of life circumstances, while lab tests like the Trier Social Stress Test (TSST) [12] provoke immediate stress responses. Considerations in choosing stress measures include stressor timescale, response characteristics, and exposure attributes [13]. Stress-related biomarkers, though elusive, offer objective indicators of physiological processes and are integrated into models explaining the stress–health relationship. Both psychological and physiological stress responses vary within and between individuals, influenced by socioeconomic, cultural, genetic, developmental, and health factors [14]. Advanced statistical models help understand variability in stress responses, which is crucial for identifying reliable biomarkers and interpreting their role in stress-related health outcomes [15]. With the rise of technology, especially the growth of the Internet of Things (IoT) and artificial intelligence, researchers have started creating models that can detect stress by looking at how our bodies react. In the early 2000s, they began exploring whether wearable devices could spot signs of stress [9]. Initial efforts focused on developing person-independent models capable of classifying stress-related signals regardless of the individual they originated from. This approach aligns with the early practices in many fields of artificial intelligence. However, the need for personalized models to capture and utilize individual variations in fine-grained physiological responses quickly emerged. While the field of personalized stress detection is evolving rapidly, it currently lacks a comprehensive review that consolidates the studies dedicated to creating personalized models. This scoping review aims to provide an overview of the models used for personalized stress detection and the available datasets for training. Simultaneously, it seeks to map non-invasive commercial wearable devices (smartwatches, bands) capable of collecting signals and providing raw data to researchers. Furthermore, the review aims to identify the implementation challenges of personalized stress detection systems in real-world contexts.

Specifically, the review will be guided by the following research questions:

RQ1: What are the primary biosignals provided by wearables that can be utilized for personalized stress detection?
RQ2: What are the key artificial intelligence (AI) techniques used to develop personalized stress detection models?
RQ3: Are there publicly available datasets for training personalized stress detection models?
RQ4: What are the wearable devices available on the market that allow the acquisition of raw data?
RQ5: What are the primary challenges encountered in the practical implementation of stress detection models in the real world?

The review is structured into three main sections. First, the Methods section discusses the approach employed to identify the literature corpus. It provides insights into the methodology used for gathering and selecting relevant studies. The second section, Results, offers a comprehensive overview of the studies that have been identified. These studies collectively contribute to addressing the research questions mentioned earlier, and this section will provide a synthesis of the findings in a coherent and organized manner. The third and final section, Discussion, is where we delve into a detailed analysis of the results derived from the review, highlighting its limitations and proposing future research directions.

## 2. Methods

### 2.1. Study Design

We conducted this scoping review in adherence to the Preferred Reporting Items for Systematic Reviews and Meta-Analyses extension for Scoping Reviews (PRISMA-ScR) statement [16]. Employing a comprehensive search strategy aimed at ensuring replicability, reliability, and transparency, we followed Arksey and O’Malley’s five-stage approach to scoping reviews [17]. This involved identifying the research question, searching for relevant studies, the study selection, charting the data, and collating, summarizing, and reporting the results.

### 2.2. Sources and Search Strategy

Studies were identified through the major academic databases (Scopus, IEEE Xplore, and PubMed), considering papers published from 2010 to 2023. Google Scholar, arXiv, and medRxiv have also been considered secondary sources for identifying new trends. The database search was completed on 31 December 2023. A standard web search using Google was carried out to identify commercial wearable devices capable of providing raw data, contacting manufacturers in cases where specifications were not available in the public materials (websites, datasheets, and software development kits). The exact queries of web search are not provided because they lack reproducibility. However, we adopted a systematic approach of examining the first 250 results of a query containing the name of the biosignal and the term “wearable device”. The keywords used for the database search instead are summarized and categorized by research question in Table 1.

### 2.3. Selection of Studies

In designing our search strategy, we meticulously adhered to predefined inclusion criteria to ensure the relevance and reliability of the gathered information. The inclusion criteria listed below served as a robust framework, guiding our pursuit of the pertinent literature to meet the specific parameters essential for our research objectives:

- The study was conducted from 2010 onwards. (We selected 2010 in accordance with the history of artificial intelligence [18], which marks its exponential growth during that period).
- The study was conducted in laboratory settings or real-life contexts.
- The study was published in English.
- The study used mainly non-invasive (the term non-invasive refers to a device that does not cause physical discomfort to the subject) wearables or bands.
- The study focused on mental stress.
- The study involved creating models or datasets for personalized stress detection or investigating the challenges in real-world applications.
- The study was not a review article.
- The study did not use synthetic data produced by Generative Adversarial Networks (GANs) or other generative systems.
- The study was not exclusively published as an abstract or poster at a conference.

Furthermore, to ensure a robust and reliable screening process, M.B., S.P., M.D., and S.G. independently conducted the screening, resolving discrepancies through collaborative discussions and consensus building. In the initial phase, studies were selected based on information from titles or abstracts. In cases where this information was insufficient, the full texts were retrieved for a more comprehensive analysis to determine eligibility. Subsequently, the full texts of all selected studies were obtained at a later stage to guarantee the quality and relevance of the information included in the review. A detailed view of the screening steps and the number of studies selected at each stage is provided in the Section 3.

## 3. Results

In this review, out of 108 screened articles, 56 were included. Specifically, we included 32 papers on developing personalized stress detection models, 13 on datasets containing raw biosignals for personalized stress detection, and 11 related to challenges for stress detection in real-world settings. Among the 56 papers, 30 were journal articles, 22 were conference papers, 3 were preprints, and 1 was published online on the university lab website. Most of the studies that focused on developing models for personalized stress detection were centered around stress detection in controlled laboratory environments (n = 14), while 13 studies focused on stress detection in real-life situations, and only 5 concentrated on controlled scenarios. The average sample size in these studies was 40.2 (SD = 60.1) subjects and gender representation was not always even, with only 2 out of 32 studies providing adequate balance.

**Figure 1:**
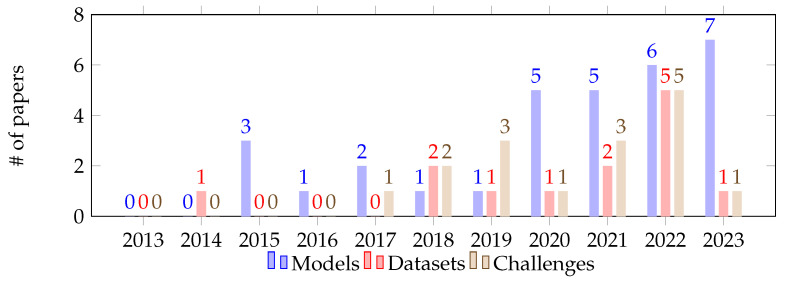
Number of studies published in each category over the past decade.

**Figure 2:**
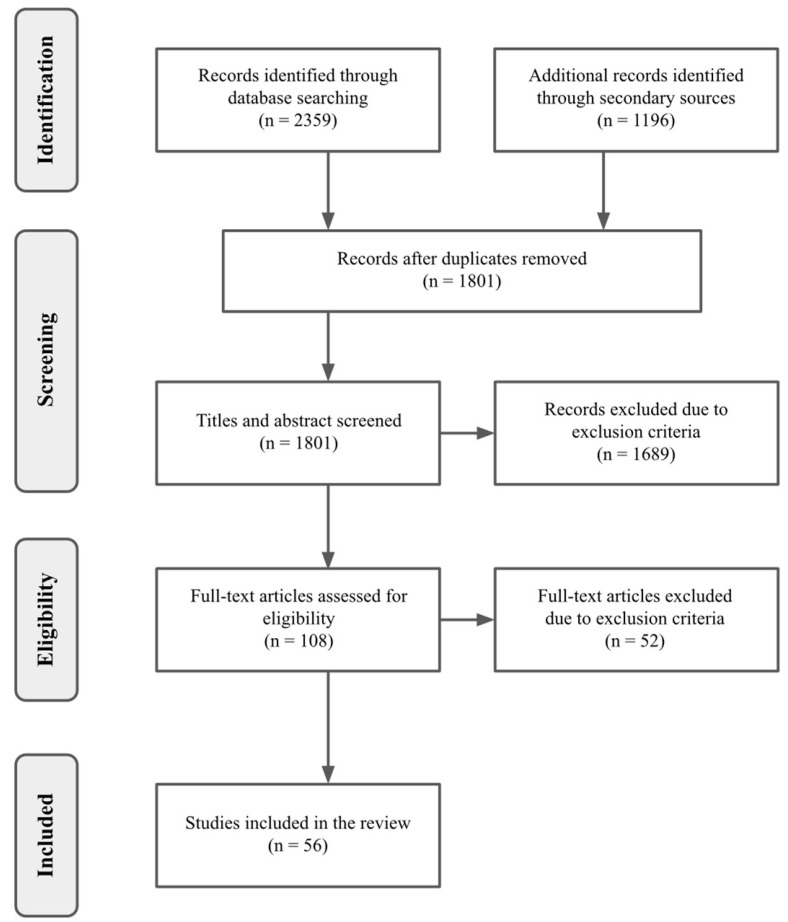
PRISMA flowchart of this scoping review.

Regarding the number of studies on personalized stress detection models published in the analyzed time span, there has been an increasing trend over the years, indicating a growing interest in personalized models (see Figure 1). Details on the selection process can be found in the PRISMA flow diagram reported below (Figure 2), while the complete list of studies included in the review is available in Section 3.1.

**Figure 3:**
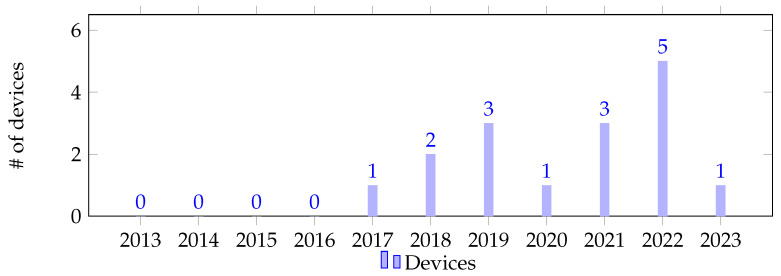
Number of devices capable of capturing raw biosignals released over the past decade.

In terms of datasets available for training personalized stress detection models, a total of 13 have been identified. Similar to the studies, these datasets have been designed to address various tasks and scenarios. Among the identified datasets, the majority (n = 9) were generated under controlled laboratory conditions, while a limited number represented controlled scenario conditions (n = 2) or real-life contexts (n = 2). As observed in the studies, there is a clear interest in the topic but, in this case, the publication flow is more irregular (refer to Figure 1). This could be attributed to the high complexity and precision that publishing a dataset requires. Further details regarding the datasets, their specific attributes, sample sizes, and labeling methodologies will be elaborated in Section 3.2. In terms of wearable devices capable of capturing raw biosignals, we have identified 16 devices. These include six wrist-worn wearables comprising four smartwatches and ten bands. Analyzing the release dates of these devices, it becomes evident that the availability of such devices has increased in the post-pandemic period (see Figure 3). A comprehensive list of these devices, along with their associated costs and features, will be elaborated in Section 3.3.

### 3.1. Biosignals and Techniques for Personalized Stress Detection with Wearable Devices

Table 2 presents a comprehensive summary of the selected studies pertinent to Research Questions 1 and 2 (RQ1 and RQ2), focusing on the use of biosignals and techniques for personalized stress detection.

#### 3.1.1. Biosignals

Before proceeding with the description of the findings in the literature, it is advisable to provide a definition of biosignal. A biosignal refers to any signal originating from a biological structure that can be measured over time [56]. Biosignals can vary in nature and contain information about the system, organ, and process that generated them. Therefore, biosignals are used to assess individuals’ health status and cognitive processes [57]. Biosignals can have different origins (electrical, pressure, chemical) and are measured using sensors, transducers, and actuators [58]. The literature analysis identified a comprehensive set of eight biosignals, detectable through wearable devices, that are well-suited for personalized stress detection. These biosignals, namely Electrodermal Activity (EDA), Photoplethysmogram (PPG), Electrocardiogram (ECG), Peripheral Skin Temperature (SKT), Respiration (RSP), Electromyogram (EMG), Electroencephalogram (EEG), and Non-Invasive Blood Pressure (NIBP), play a crucial role in reflecting psychophysiological changes induced by the sympathetic nervous system. When exposed to stressors, these biosignals mirror the intricate interplay of physiological responses orchestrated by the sympathetic nervous system. The body’s reaction to stress involves the activation of the sympathetic nervous system, triggering the release of stress hormones such as adrenaline, an increase in heart rate, and preparation for immediate action—a response commonly known as the “fight or flight” mechanism. This heightened state of alertness is accompanied by muscle tension, changes in metabolism, and other physiological changes. Significantly, these stress-induced reactions extend beyond high-stress situations and manifest in more commonplace scenarios such as traffic, workplace pressure, and financial concerns. Consequently, even in everyday circumstances, wearable devices can capture and reveal the individual’s stress through these biological signals [59]. Notably, the intensity of the physiological response may vary, and this fluctuation is influenced by the severity of the stressor [60]. To provide a more detailed understanding, Table 3 presents the number of studies using each biosignal, along with brief definitions and their specific relationships with stress. Another aspect that emerged from the literature concerns the number of sources (biosignals) used to train the models. More than half of the studies adopted a (multimodal) multi-sensor approach (n = 19). The multimodal approach seems to generally improve performance compared to single-source models, indicating the capability of multi-sensor systems to capture diverse physiological responses associated with stress [28,39]. Among the studies using a single biosignal, the majority focused on PPG/ECG (n = 10) and EDA (n = 3). These biosignals produce comparable results in a single-modality setup [28,29], which is particularly interesting since PPG/ECG sensors are now available on many commercially available smartwatches, highlighting the potential real-world impact of personalized stress detection studies. Finally, some studies (n = 11) have addressed the issue of the impact of individuals’ motion on biosignals by incorporating motion information into the models. Specifically, readings from accelerometers (ACC) (n = 11) and gyroscopes (GYR) (n = 1) of wearable devices have been employed. The joint use of such information along with biosignals allows for their cleaning [28,29,36,61,62] and makes it possible to adapt models to different contexts, providing them with context awareness. This enables the proper functioning of the models even in dynamic situations and facilitates the discrimination of mental stress from physical stress. In cases of physical stress, individuals may display cardiac or sweating patterns similar to those observed in stress states, but these are due to physical activity. The presence of physical activity is clearly visible in the data captured by accelerometers and gyroscopes, allowing the models to accurately differentiate between the two types of stress [61,62].

#### 3.1.2. Techniques

**Figure 4:**
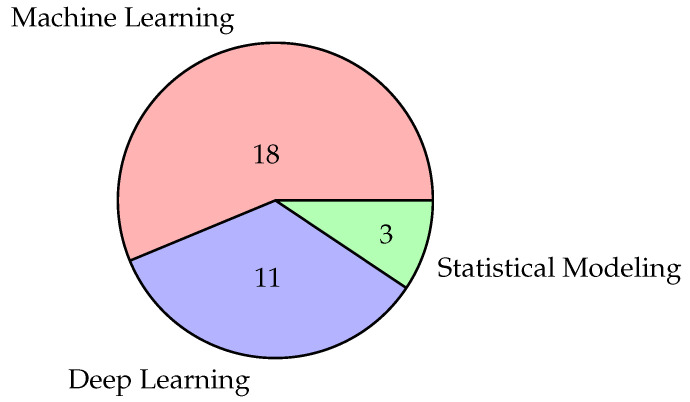
Number of studies grouped by AI approach.

Regarding the techniques for developing personalized stress detection models, the search identified three main approaches: a statistical approach, a machine learning approach, and a deep learning approach. To date, the most prevalent approach involves traditional machine learning methods, followed by deep learning architectures, and, finally, statistical techniques (see Figure 4). Before delving into various approaches, it is necessary to provide a preamble to better frame the personalized stress detection task. Regardless of the adopted approach, all the approaches mentioned use individual subject data to develop personalized models. In other words, according to this problem definition, each subject poses a distinct classification or regression problem from other subjects. Consequently, the train–test split is performed on individual subject data, which are used respectively to train the model and evaluate the performance of the specific subject. It should also be explicitly stated that our research did not find fully unsupervised methods. Therefore, everything reported should be considered limited to signal classification through supervised methods or methods that require at least a minimal amount of labeled data.

##### Machine Learning Approach

The machine learning approach involves training algorithms on labeled datasets to enable the automated identification and categorization of stress levels based on input features, facilitating accurate and efficient stress assessment. Nearly all studies follow a classic pipeline consisting of multiple steps: signal preprocessing, feature extraction, algorithm selection, feature selection, and hyperparameter tuning. This approach allows for interpretable models but requires a clean signal and a feature engineering process based on prior knowledge. In other words, in this approach, the classifier’s accuracy depends on the quality of the signal and the relevance of features extracted from it. However, raw signals extracted from the sensors contain artifacts due to motion, electromagnetic interference, and other disturbances from the surrounding environment. For this reason, more than half of the machine learning approach studies (n = 13) have introduced strategies for cleaning the signal based on filters.

In signal processing, a filter is a mathematical operation or a device that modifies or extracts certain features from a signal, often used to remove unwanted components or emphasize specific characteristics. In particular, the studies included in the review used lowpass, highpass, bandpass, and notch filters. A summary of the filters used, a definition of each filter, and related biosignals are reported in Table 4. In relation to machine learning algorithms, a variety of classical supervised algorithms and specific combinations of unsupervised and supervised techniques are used. A comprehensive list of the identified algorithms is delineated in Table 5.

As observed in Table 5, a preference for tree-based models is evident (n = 9). Tree-based models prove especially advantageous in medical settings due to their interpretability and capacity to reveal meaningful patterns. These models facilitate the assessment of feature importance and the visualization of rules utilized for classification, thereby enabling domain experts to inspect and evaluate the validity of the classification model visually. Furthermore, studies included in the review that compared tree-based models with other algorithms highlighted superior performance of the tree-based solutions [21,28,29,38], affirming the effectiveness of such models in medical applications and, in particular, in personalized stress detection.

In terms of extracted features, the studies did not use a predefined set of features. Instead, researchers derived several aspects from biosignals based on the literature and prior knowledge. However, a significant number of features were consistently considered across multiple studies. Table 6 lists the most common features for each signal identified in the selected studies, providing a brief definition of each feature and a reference to studies that used that specific feature. If analyzed with attention, Table 6 highlights a particularly interesting aspect: many features extracted from PPG and ECG signals measure the same phenomena (e.g., parasympathetic activity). This aspect should be considered when developing machine learning models, as incorporating multiple features measuring the same phenomenon introduces noise into the model. A solution researchers propose involves implementing a feature selection step to remove redundant variables. However, this aspect is underestimated, as only 5 [21,24,25,29,49] out of 18 studies have included some form of selection of the most relevant features.

##### Deep Learning Approach

The deep learning approach is an approach based on learning through the use of sophisticated neural networks. Specifically, it employs layers to extract increasingly high-level features from raw data. In contrast to the machine learning approach, here, there is no need to extract any features from the input signal, and the performance depends on the researcher’s skill in defining the most suitable network architecture to solve the problem. Unlike classical machine learning models, this approach generates black box solutions whose interpretability for the final user is limited or requires specific techniques such as activation maps. The studies on personalized stress detection with deep learning are limited (n = 11). However, they can still contribute to outlining the emerging components of architectures and the strategies researchers employ to address specific problems. Discussing the particularly relevant components of the architectures introduced by researchers, we find the Convolutional Neural Network (CNN) layers [26,45,47,50,51] and self-attention mechanisms [39]. CNN layers (used in 7 out of 11 studies) are fundamental building blocks of a CNN. A CNN layer consists of a set of learnable filters (called kernels) that slide over the input data to perform convolution operations. The convolution operation involves element-wise multiplication of the filter weights with the input data, followed by summation. This process allows the network to learn hierarchical representations of features in the input data automatically. In addition to the convolutional operation, a CNN layer typically includes an activation function (e.g., ReLU) to introduce non-linearity and a pooling layer for down-sampling, reducing the input data’s spatial dimensions.

**Figure 5:**
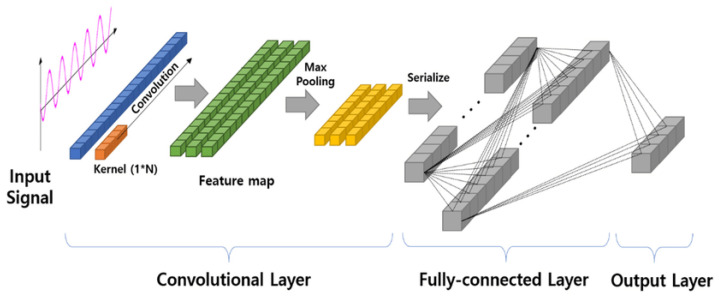
1D CNN in signal processing. Adapted from [78].

**Figure 6:**
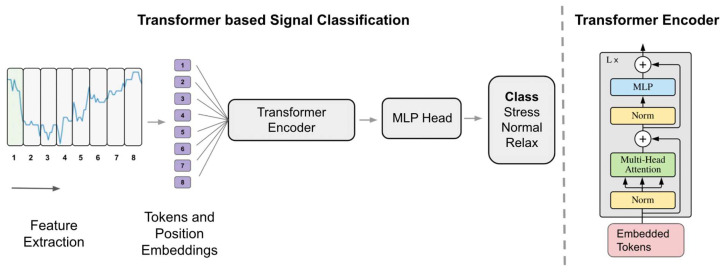
Transformer-based architecture for signal classification. Adapted from [79].

The studies [26,45,47,50,51] included in the review used a variant of CNNs known as a 1D CNN. In a 1D CNN, filters slide over the input sequence to perform convolutional operations (see Figure 5). The sliding-window convolutional operations in a 1D CNN facilitate the network in recognizing patterns and dependencies across different time scales. For instance, in stress classification, they can effectively identify sudden changes in heart rate or variations in skin conductance that are indicative of stress responses and consequently extract relevant features automatically for the final classifier [50]. However, while 1D CNNs excel at processing data in short time windows, they have limitations in learning long-range dependencies within the signal. To address issues arising from long-range dependencies, especially in very wide time windows, researchers have proposed the use of Long Short-Term Memory (LSTM) [33,35,44], and Transformers [39] for solving stress classification tasks. LSTM networks [75] are a type of Recurrent Neural Network (RNN) specifically designed to address the challenge of capturing long-range dependencies in sequential data. Unlike traditional RNNs, LSTMs can learn and retain information over extended time intervals, making them well-suited for tasks such as signal classification [76]. This is achieved through a specialized memory cell consisting of a cell state and three gates: input gate, output gate, and forget gate. The cell state allows LSTMs to store and access information over long periods while the gates regulate the flow of information into and out of the cell, enabling the network to manage and preserve relevant information for the task. In stress monitoring, physiological signals like heart rate variability require analysis over longer durations to accurately discern stress patterns. LSTMs handle this by maintaining a state that can remember and relate past information to present data, which is crucial in environments where stress indicators manifest intermittently and evolve over time [33,35]. On the other hand, Transformer [77] is a type of deep learning architecture designed to classify feature vectors by employing self-attention mechanisms. These mechanisms allow the model to dynamically assess the importance of various elements within the input sequence (See Figure 6).

In stress detection tasks [39], the process starts by segmenting continuous data streams into smaller segments and then extracting relevant features from these segments. To acknowledge the order of the data, positional encoding is added to introduce temporal context. An attention mechanism is utilized to weigh the importance of different elements within the biosignal data, capturing their relationships. Finally, the transformer architecture is completed with a classification head. Focusing on interesting strategies related to deep learning, the use of self-supervised learning (SSL) is highlighted. Before delving into the details of self-supervised learning, it is essential to note that, in the field of personalized stress detection models, it is common practice to collect real-life data using wearable devices. Wearable devices enable data collection in ecological situations. However, a tradeoff must be found between the demand for labels from participants and the quantity of data needed to develop an accurate model. To address this issue, researchers have started collecting a limited number of labels (in the analyzed studies, approximately four per day) while simultaneously passively recording unlabeled biosignals. The underlying idea of this data collection is to leverage the knowledge contained in unlabeled signals through self-supervised learning techniques. Self-supervised learning involves training models on unlabeled data to build background knowledge and mimic a form of common sense in AI systems. The workflow of self-supervised learning comprises two steps: pre-training on unlabeled data to build background knowledge and fine-tuning on downstream tasks. Pre-training involves training a model on data without annotations, using a pretext task to guide the model to learn intermediate data representations. In contrast, downstream tasks are specific tasks to which the knowledge gained from the pre-training phase is transferred. Studies using this method [47,50] learned a representation of biosignals during the pretext phase without labels. They then fine-tuned using labels from the downstream task to predict stress. In terms of performance, both studies showed more stable results [47,50] than a CNN baseline using a fully supervised approach. However, only [50] demonstrated a clear improvement in terms of accuracy. The study [50] also found that self-supervised learning can achieve performance similar to fully supervised approaches using less than 30% of the data. These initial findings are promising and suggest the need for further investigation.

##### Statistical Approach

The statistical approach relies on a more traditional signal analysis using statistical algorithms. The strengths of studies employing this approach lie in the interpretability of the rules, signal classification speed, and the ability to adapt the classifier over time. Like machine learning, this approach heavily depends on the signal selected to identify stress and quality of such signal. For this reason, all the studies utilizing the statistical approach included in the review employ signal filtering methods based on motion detection [23] and filters [43,52]. Regarding the signals examined in the studies, two focused on EDA [23,52], while one considered PPG [43]. In general, the pipeline employed by studies based on statistical approaches can be summarized in two main steps, with an optional third step: collecting a baseline (composed of filtered biosignals) for the subject, constructing rules through statistical algorithms, and potentially adapting these rules on the fly over time. Two of the studies employ particularly interesting algorithms. Ref. [43] uses an adaptive referencing range expectation maximization (AR-EM) algorithm. AR-EM is a method employed in biosignal analysis to generate personalized adaptive reference ranges. It employs an expectation-maximization (EM) framework to efficiently estimate unknown parameters and optimize reference ranges based on an individual’s records. Meanwhile, Ref. [52] utilizes a MOS algorithm [53] variant to compute an individualized MOS score for each second in the measurement signal. This score is calculated by assigning values based on the amplitude and slope of the EDA signal and following the distribution of the signal. To date, no study has compared the effectiveness of statistical approaches with other techniques, and their application in unstructured real-life contexts has yet to be explored.

### 3.2. Datasets for Personalized Stress Detection

The literature review identified 13 public datasets (summarized in Table 7) suitable for developing personalized stress detection models. All identified datasets, except one [43], are multi-signal and contain information from multiple sensors. The majority of the datasets (n = 9) were developed under laboratory conditions, while only a small number of datasets were created in controlled scenarios (n = 2) and real-life conditions (n = 2). The average sample size is 42.3 participants, ranging from a minimum of 10 subjects to a maximum of 120. The participants’ average age is 25.2 years. These two pieces of information suggest both a limited size and the presence of age bias within most of the datasets. However, it should be noted that one particular study [80] provides a generous and representative sample (120 subjects) of the French population. In 8 out of 13 studies, a strong gender bias is also present. All datasets, except one [81], focused on the healthy population. Among the most included signals in the datasets, we found EDA (n = 12), PPG (n = 11), ECG (n = 7), SKT (n = 6), and ACC (n = 6). Ref. [31] appears to be the most popular dataset, with 25% (n = 8) of the studies included in the review relying on it. Its popularity may stem from several factors: it utilizes a solid protocol and contains multimodal data from both the chest and wrist locations, allowing for comprehensive comparisons and benchmarking. Additionally, it is easily accessible and its data are organized in a very convenient way with already synchronized labels. These features make it exceptionally user-friendly and functional for researchers aiming to study stress under different conditions.

Regarding the assignment of ground-truth stress labels, more than half of the studies (n = 7) used at least one validated tool. Among the most commonly used tools are the NASA Task Load Index (NASA-TLX), the State Trait Anxiety Inventory (STAI), and the Self-Assessment Manikin (SAM). NASA-TLX [90] is a subjective workload assessment tool used to evaluate perceived workload across different tasks. It provides a multidimensional rating of mental demands, physical demands, temporal demands, performance, effort, and frustration. STAI [91] is a self-report questionnaire that assesses two types of anxiety: state anxiety and trait anxiety. State anxiety refers to the individual’s current emotional state, reflecting temporary feelings of anxiety and stress in a specific situation. Trait anxiety, on the other hand, measures a person’s general tendency to perceive situations as threatening. SAM [92] is a tool designed to assess subjective reactions to various stimuli, including emotional responses such as valence, arousal, and dominance. It typically involves using graphical representations, such as stylized human figures or “manikins” to allow individuals to express their emotional states along these dimensions. It is important to emphasize that although these tools are validated, the constructs they measure may only partially overlap with stress and may introduce distortions in the labels. In terms of non-validated instruments, n-point Likert scales are used to assess perceived stress. In datasets that focus on real-life contexts, the primary method for assigning ground-truth labels is based on surveys. As seen in many articles that covered real-life experiments [32,36,38,39,42,47,54], a dataset [88] provides labeled biosignals through Ecological Momentary Assessments (EMAs). EMA is a research method used in psychology and related fields to assess individuals’ real-time experiences, behaviors, and physiological states in their natural environments [93]. Unlike traditional assessment methods that rely on retrospective recall, EMA involves collecting data at multiple points in time, often using electronic devices such as smartphones or wearable sensors by sending prompts or surveys through them. This approach might be seen as invasive, but if the number of labels is limited, it can provide good quality ground truth for signal classification.

The analysis of stressors, presented in Table 8, has highlighted the adoption of two main types of stressors used for building the datasets. The first type of stressor pertains to daily life stressors that mimic situations individuals may encounter in real-life contexts, while the second includes artificial procedures for eliciting stress states. Given the predominant presence of datasets developed in laboratory settings, there is a notable tendency toward using artificial procedures for stress state elicitation. In fact, 7 out of 13 datasets incorporate at least one of these procedures. While utilizing controlled procedures to evoke stress states allows for enhanced control over the dataset, it also comes with the risk of not producing information suitable for developing personalized stress detection models that work in the real world. In fact, despite the proven effectiveness of controlled procedures in inducing stress responses within a laboratory environment, they may need more direct evidence of ecological validity [94]. Although most datasets used either daily life stressors or artificial procedures, only three studies [80,84,89] implemented both strategies. To conclude, there are currently several datasets available for the development of personalized stress detection models; however, most of them have been developed in a laboratory setting and may not ensure model transferability to the real world. Additionally, these datasets exhibit some biases related to the age and gender of participants. Therefore, there is a need to develop datasets in naturalistic contexts with less distorted sample characteristics.

### 3.3. Devices for Raw Data Collection in Stress Detection Research

The research on devices has identified a total of 16 devices for acquiring raw signals. More than a third of the identified devices (n = 6) allow the collection of information from multiple biosignals, and almost all of them (n = 14) collect movement-related data, suggesting good potential for developing real-world applications. Regarding the wearability of the devices, a heterogeneous picture emerged: of the identified devices, six are wrist-worn, four are worn on the arm, two on the chest, and two on the forehead. Additionally, two devices can be worn in different parts of the body based on the biosignal to be measured. Regarding battery life, different performances have emerged, ranging from a minimum of 4 h for the EmotiBit to a maximum of 16 days for the Polar H10. Concerning the possibility of interfacing devices with mobile systems, all devices were potentially compatible with Android, while only less than half with iOS (n = 7). The most prevalent protocol for communication with wearable devices is Bluetooth, supported by all devices included in Table 9. To date, despite the great potential, the possibility of extracting raw data from commercially available smart rings is not available. In general, the devices available on the market today exhibit a high degree of wearability and enable the capture of a wide range of biosignals. The cost involved in purchasing them is also reasonable and within reach even for research groups with limited resources. However, notable challenges persist, primarily concerning limited battery life across most of these devices. Additionally, seamless integration with iOS devices, which constitute a significant share of the total device market in certain regions, remains a considerable hurdle.

### 3.4. Real-World Challenges in Stress Detection Solutions

Our search on the real-world challenges in stress detection solutions identified 11 articles. A qualitative examination of these articles unveiled five prominent themes: data quality and distortions, technical aspects related to wearable devices, user experience and behavior, privacy, and interpretability. On a quantitative level, the most frequently encountered theme is data quality and distortions (n = 9), followed by user experience and behavior (n = 4) and technical aspects related to wearable devices (n = 3). On the other hand, the themes of privacy and interpretability were addressed by just one article each (See Table 10).

#### 3.4.1. Data Quality and Signal Distortion

Concerning data quality and signal distortion, three main issues have been identified: signal distortion, label assignment, and missing data handling. Regarding signal distortion, studies have emphasized that raw data from wearable devices is often distorted and might not meet minimum standards for their use in real-world contexts. Among the most common causes of these distortions are motion artifacts [113,114,116,118], physical or human activity [111,116,118,119], and optical sensor issues [113]. Motion artifacts and human activity artifacts stem from the subject’s movement during measurement, where the sensor may lose adherence (in the case of ECG or EDA) or move too far from the skin (in the case of PPG), resulting in instances of poor contact and, in some cases, complete loss of contact. While this poses a tangible problem, the literature suggests strategies for managing such distortions, including the incorporation of accelerometer data into models and the implementation of human activity recognition classifiers for signal cleaning, as well as the use of filters discussed in Section 3.1.2. Regarding optical sensor issues, the situation is more complex but limited to PPG and SKT sensors. These sensors rely on skin characteristics for signal construction and are consequently strongly influenced by them. Specifically, tattoos in the wrist area, skin perfusion, and skin tone may distort the optical sensor output due to different reflection patterns [113]. The solution to these issues is unclear and represents a more concrete obstacle than motion artifacts. While signal distortion may adversely impact the integrity of data used for developing and implementing personalized stress detection models, we also confront the issue of label distortion. The quality of labels in a dataset is crucial as it guides the algorithm in learning. However, the quality of labels poses a particularly complex challenge in developing stress detection models, as it is necessary to find a strategy for measuring a vague construct such as stress. The definition of stress as a psychological construct is still an ongoing process, and researchers have not yet reached a consensus. The challenge, therefore, is to reliably measure a construct that is not well-defined. One solution proposed by researchers is to expose individuals to protocols that elicit stress states and use cortisol tests for label assignment [117]. However, besides lacking ecological validity, this approach is impractical for creating personalized stress detection models in the real world, as it would require a time-consuming in-person session to create the training set, limiting the scalability. An alternative approach researchers propose is to use validated scales that measure constructs ideally close to perceived stress for label assignment. Although this solution is more methodologically robust, it should be emphasized that consistency between the scale and model output has to be maintained, and any data manipulation or aggregation (for example, for building a binary classifier from scales’ continuous values) should be avoided [117]. The quality of labels thus represents an open problem that requires clarification both regarding the construct measured and the instruments for its measurement, which should be compatible with personalized stress detection solutions that are implemented. As previously outlined in the section about signal distortion, real-life data from wearable device sensors may contain missing data [117]. Specifically, missing data in biosignals represent interruptions in the time series. The presence of gaps in a time series poses a practical problem, affecting the feature extraction stage in machine learning-based approaches and the possibility of utilizing such biosignal fragments in deep learning models. Interpolation was traditionally employed to address this issue; however, this approach is outdated, may introduce bias, and can adversely affect model performance. Recent studies have proposed two main methods to manage the gaps in the time series. The first method [115] can be applied in developing personalized stress detection models based on machine learning and involves using complete biosignal fragments to train classifiers capable of predicting the missing features of incomplete signals. This approach introduces fewer distortions than interpolation because it operates not on the time series but on its high-level informative content, leveraging globally learned knowledge. The second method, on the other hand, is more general and models signal fragmentation as a sparsity issue. In particular, according to [112], it is possible to exploit nuclear norm minimization, a method based on the low-rank assumption, to derive unobserved sensor data thanks to mathematical properties (i.e., observed entries are sampled uniformly at random, and exact recovery can be achieved leveraging probability). Although these approaches are effective, most studies developing personalized stress detection models in real-life scenarios tend to discard incomplete signals or rely on interpolation to reduce computational load and model complexity. Considering this, it is essential to examine new perspectives for managing missing data, ensuring a seamless, efficient, and prompt resolution of data gaps.

#### 3.4.2. Technical Aspects Related to Wearable Devices

Focusing on the technical aspects related to wearable devices, the literature analysis has identified three limitations that affect real-world implementations of personalized stress detection models: battery life [111,116], computing and network capabilities [111], and vendor updates [120]. Battery life is the primary challenge when deploying personalized stress detection models that rely on biosignals from wearables. While the production processes of wearable devices have improved over the years, resulting in increasingly efficient processors, the physical limit of batteries remains. Most commercially available wearable devices, especially smartwatches, use batteries with capacities below 500 mAh [122], capable of supporting device activity for only a limited number of hours. The battery constraint impacts the operational choices researchers are forced to make. Specifically, researchers must find a tradeoff between information granularity and battery life [111,116]. Hitting the battery limit can result in incomplete data and study dropouts due to poor experience. In order to reduce battery drain, researchers may limit the number of measurements, data upload frequency, and the complexity of on-device operations. Regarding on-device operations, it is essential to note that wearable devices offer limited network and computing capabilities and are currently unable to execute complex models. These functions are usually outsourced to an edge layer (e.g., phone) and cloud solutions [111]. Lastly, it is noteworthy that most wearable devices lack open-source accessibility, which introduces potential challenges associated with the code governing the sensors. Researchers have raised concerns about the possibility of encountering alterations in raw data stemming from updates or firmware modifications to the device introduced by vendors. Addressing these technical challenges is critical and involves implementing strategies for optimizing processes or data collection, exploring new hardware solutions, and granting researchers greater control over the source code of devices, potentially through the adoption of open-source solutions.

#### 3.4.3. User Experience and Behavior

Regarding user experience and behavior, three main challenges have been identified for implementing real-world solutions for personalized stress detection: the presence of technical support, granting user data access, and the implementation of best practices for EMAs. Regarding the technical support side, researchers have emphasized the need to establish a contact line with the participants involved in the study [114,121], as it is common for them to encounter difficulties that, if ignored, can lead to missing data or study dropout. In particular, the presence of technical support helps to reduce device malfunctions due to improper usage and to manage any configuration issues [114,121]. However, it should be noted that a user-friendly wearable like a consumer smartwatch reduces the need for frequent intervention. Regarding user data access during the study, it has been observed that, although it can positively affect motivation, it can lead to behavior manipulations by the participant, which may change his or her behavior to observe effects on the dashboard provided [114]. It should be emphasized, however, that this behavior was observed in a sample of adolescents, so it may be more relevant to specific types of user groups. Considering best practices for the implementation of EMAs, researchers largely agree on the need to identify an accuracy/labels tradeoff that allows collecting a sufficient number of labeled data through EMAs for creating a training set that is large enough to train a good classifier for stress [120,121] without posing a significant burden on the subject. While exaggerating with labels could lead to dropout, having a small amount of data points could negatively impact the final performance of the classifier. Identifying a strategy to optimize the number of EMAs is therefore crucial. Findings from [116,121] suggest that user response rates are influenced by the activity performed at the time of survey reception, the time of day, the subject’s mood, and personal patterns. Ref. [121] suggests that developing machine learning models for identifying the ideal timing for the user to send the minimum number of communications while still collecting the desired amount of data would be beneficial. Finally, engaging solutions (e.g., apps) for collecting EMAs seem to promote higher response rates than more traditional methods (e.g., SMS). In conclusion, researchers should focus on building tailored solutions to gather the data, considering the need for technical support and implementing strategies for collecting training labels with a less invasive and more engaging approach.

#### 3.4.4. Privacy

Privacy refers to the right of individuals to keep personal information and activities confidential. Given that solutions for personalized stress detection are based on physiological data and contain sensitive information about the psychophysical state of an individual, this dimension becomes significant. Medical data and privacy are critical aspects of healthcare, and protecting patients’ sensitive information is a legal and ethical responsibility. In this regard, researchers [120] proposed a set of best practices for managing data in studies or real-life applications. In particular, according to privacy by design principles, participants should be informed in detail about the data collection process, the use of collected data, and the purpose of the research. Data collection should also be limited to essential data, excluding those not strictly necessary for the stress detection task, such as GPS data, which could indirectly identify an individual. To address the risks of data leaks and promote privacy, it is advisable to integrate solutions that adhere to security standards and allow users to limit or revoke data access at any time. A potential strategy to mitigate privacy-related risks could be developing personalized stress detection models that work on wearable devices [41]. However, it is essential to remember that, with current technology, wearables have limited computational power and batteries that limit this possibility. Regarding the legal aspect of implementing models in real life, many countries are developing regulations or guidelines to guide the adoption of privacy-friendly AI. However, a shared set of rules has yet to be identified. Fostering a dialogue between developers, end-users, and regulatory bodies is pivotal to establishing a shared understanding of the ethical considerations involved in personalized stress detection model development. As we continue to advance in this field, a commitment to responsible innovation and a holistic approach to privacy will be crucial in shaping the future of research.

#### 3.4.5. Interpretability

As mentioned earlier, the stress detection task falls within medical applications and, as such, requires models capable of providing information about the rules followed by the model in making decisions. A white box model is preferable in medical AI due to its transparency, providing clear insights into the decision-making process, which is crucial for ensuring accountability and trust in healthcare applications. This transparency allows medical practitioners to understand and interpret the model’s decisions, enhancing usability and facilitating collaboration between clinicians and AI systems. In this regard, researchers [118] have proposed using SHapley Additive exPlanations (SHAP) values to make the models more interpretable. SHAP values fairly distribute the contribution of each feature to the prediction of a model among its individual features. By assigning a numerical value to each feature’s impact on the prediction, SHAP values enhance interpretability by revealing the importance of different factors in influencing the model outcomes. This helps to understand the model’s decision-making process better but also aids in explaining individual predictions, making the model more transparent and trustworthy for users and stakeholders. However, the limitation of this solution lies in the fact that it can be mainly applied to models that adopt a machine-learning approach. The explainability of deep learning models is a more complex topic that requires targeted investigation by researchers. So far, interpretable models for personalized stress detection based on deep learning have yet to be available, and more research on interpretable deep learning models is needed.

## 4. Discussion

Understanding and managing stress is a fundamental aspect of human existence, with its roots in discomfort and mental tension triggered by life’s challenges. How we navigate stress plays a pivotal role in shaping our overall well-being. In the era of technological advancement, particularly marked by the rise of the IoT and AI, researchers have ventured into creating models that can discern stress by closely observing bodily reactions. This not only allows for continuous monitoring but also offers a cost-effective approach. While stress detection systems have become increasingly widespread, the last decade has seen a notable shift towards personalized models. These models are designed not only to capture the unique variations in individuals’ stress responses but also to offer predictions tailored for specific contexts, including but not limited to clinical settings. Existing literature reviews [9,123,124,125,126,127,128] have explored stress detection models, predominantly focusing on generalized frameworks. However, until now, none have consolidated the crucial dimension of personalized models. This comprehensive review addressed this gap by systematically answering the research questions introduced earlier. Specifically, it delved into the most commonly used biosignals for personalized stress detection (RQ1), the AI techniques employed by researchers in developing such models (RQ2), and the publicly available datasets for training personalized models (RQ3). Additionally, the review identified wearable devices in the market that facilitate the acquisition of raw data (RQ4) and outlined the main implementation challenges in developing real-world stress detection solutions (RQ5). This review uniquely provides a comprehensive mapping of existing datasets specifically suitable for training subject-tailored classifiers, a critical development for effective personalized stress detection. To our knowledge, this is the first review that focuses exclusively on personalized stress detection models, marking a significant advancement in the field. Lastly, we offer an in-depth analysis of the real-world challenges that must be addressed to deploy effective, transparent, and fair stress detection models, setting a new standard for future research in this area.

### 4.1. Biosignals

In the exploration of biosignals for personalized stress detection through wearable devices, our analysis has identified a spectrum of eight key signals: EDA, PPG, ECG, SKT, RSP, EMG, EEG, and NIBP. Notably, existing models predominantly center around the utilization of EDA and PPG signals. EDA, measuring skin conductance, reflects sweat gland activity influenced by the sympathetic nervous system during stress, while PPG records blood flow changes, influenced by stress hormones activating the sympathetic nervous system, ultimately altering heart rate and causing vasoconstriction. Some studies [28,29] revealed the potential of EDA and PPG in delivering comparable and reliable results in single-modality setups. This finding is surprising as EDA has historically been considered the gold standard for stress detection; moreover, the discriminative power of PPG gains further significance considering the widespread availability of PPG sensors in today’s smartwatches. However, the need for precision in personalized stress detection models prompts a call for attention to multimodal approaches, which leverage multiple sensors. While EDA- and PPG-only models show promise, the incorporation of additional signals, such as motion data from accelerometers and gyroscopes, emerges as a key factor in enhancing accuracy [28,29,36,61,62]. The integration of motion data not only refines biosignals for improved reliability but also facilitates model adaptation to diverse contexts, ensuring functionality in dynamic situations. This comprehensive approach aids in distinguishing mental stress from other stress types [61,62], providing a more nuanced understanding of an individual’s stress state. In conclusion, the array of biosignals available today allows for a comprehensive approach to stress detection, with sensors already widely used in smartwatches among the population. While evidence supports the effectiveness of a multimodal approach [28,39], the discussion surrounding the trade-off between the number of sensors and the improvement in accuracy necessitates further investigation. Consideration of both economic and computational costs is crucial when evaluating the implementation of multimodal models in wearable solutions.

### 4.2. AI Techniques

In discussing the techniques for developing personalized stress detection models, our exploration has unveiled three primary approaches: a statistical approach, a machine learning approach, and a deep learning approach. Regardless of the selected methodology, each approach utilizes individual subject data to construct personalized stress models, treating each individual as a distinct classification or regression task. An intriguing gap identified in our research is the absence of fully unsupervised methods for personalized stress detection, prompting consideration for label-free solutions in future studies. Currently, the prevailing trend in this field favors traditional machine learning methods, followed by deep learning architectures, and, finally, statistical techniques, with the deep learning approach gaining prominence. The statistical approach relies on traditional signal analysis using statistical algorithms, excelling in interpretability, signal classification speed, and classifier adaptability over time, but these solutions heavily depend on signal quality and rules. Machine learning models instead are trained on labeled datasets for automated stress identification based on input features, and involve steps such as signal preprocessing, feature extraction, algorithm selection, and hyperparameter tuning. While this approach allows interpretable models, it necessitates a clean signal and prior knowledge-based feature engineering similarly to statistical models. On the other hand, the deep learning approach employs neural networks to learn high-level features from raw data, eliminating the need for pre-extraction of features from the input signal. However, resulting black box solutions limit interpretability unless specific techniques like activation maps are implemented. As of today, there are also no studies comparing the performance of various approaches on the same dataset, making it challenging to assess the tradeoff between interpretability, computational load, and resulting accuracy. Another aspect introduced by some of the reviewed studies [47,50], which needs further investigation, is the use of partially labeled datasets gathered by wearable devices to train the models. Collecting a limited number of labels while concurrently recording unlabeled biosignals passively aims to extract valuable knowledge from these unlabeled signals, opening new opportunities for efficient and practical stress-related data collections and model training. In conclusion, the need for further research is evident, particularly in the use of unlabeled data and comparative studies involving state-of-the-art deep learning, machine learning, and statistical methods on identical datasets. These studies should meticulously consider factors such as computational power, inference time, and the possibility of running models on devices with limited resources (e.g., smartwatches). Additionally, there is a need for further studies in clinical practice to assess the acceptability of black box versus white box models. Legal considerations regarding the deployment of such models in hospitals and real-world clinical settings also merit careful exploration.

### 4.3. Datasets

The exploration of the literature for datasets instead revealed a generous amount of public datasets (n = 13) that serve as valuable resources for the development of personalized stress detection models. These datasets, which are rich in multi-signal data from sensors, are excellent resources for the field’s advancement. However, a close examination of these datasets reveals several significant flaws. To begin, the majority of the datasets have sample size limits and significant biases, notably in terms of gender and age demographics. Furthermore, a major amount of the collected data is restricted to controlled laboratory conditions, which may limit the applicability of stress detection algorithms to real-world scenarios. Beyond these environmental constraints, concerns are raised about the assignment of ground-truth labels through the use of some validated tools, such as NASA-TLX and STAI, which measure constructs only partially overlapping with the complexity of stress [117]. This aspect raises questions about the potential impact on model training, suggesting that the approximation of stress through these tools may introduce subtle biases into the model outputs. In other words, this approximation could potentially be inherited by the model during the training phase, influencing outputs in a manner not directly measurable by metrics but consistently impacting results in a systematic way. Furthermore, the protocols employed in developing most datasets involve artificial procedures to elicit stress states (e.g., Stroop Test, TSST). These procedures, however, fail to accurately reflect real-life situations, compromising the ecological validity [94]. To our knowledge, there is limited research focusing on this aspect, highlighting a critical gap in the literature. Further investigations into the transferability of data produced with such procedures are deemed necessary to enhance the robustness of stress detection models and ensure their applicability to real-world settings. Future efforts should aim at producing datasets with larger and less biased samples that better represent the general population, while avoiding distortions related to age or gender. Furthermore, an in-depth exploration of the relationship between measurement tools and the specific dimension of stress being investigated is essential for the development of accurate models, both in terms of raw performance and construct validity. To foster the development of models applicable in real-world settings, the creation of more datasets featuring information collected through EMAs [129] is suggested. Such datasets, capturing individual experiences in naturalistic conditions, offer a means to overcome the limitations introduced by artificial stress induction procedures and provide a more authentic representation of stress states. It is also crucial that these datasets are made more accessible and organized in a convenient way, with already synchronized labels, similar to [31], which has been shown to be a popular and user-friendly dataset for researchers. This would facilitate the development of stress detection models and encourage more researchers to contribute to the field.

### 4.4. Devices

In our focus on devices, our web search identified commercial wearables capable of capturing raw data for all the biosignals specified in RQ1. The majority of these devices enable the collection of more than one biosignal, with many also facilitating the collection of movement-related data crucial for training more effective models. Without delving into the technicalities of each wearable, we suggest researchers focus on certain features (battery life, connectivity, and compatibility) when finding and choosing the tool that best fits their research. In particular, we identified battery life to be highly heterogeneous among the devices, ranging from a few hours to a few days. Another highly relevant aspect is connectivity and compatibility with mobile devices. In this regard, Android exhibits superior compatibility, while iOS seems more closed and challenging to integrate with wearables. Bluetooth stands as the gold standard, thanks to its low-energy features (BLE) that allow the device to optimize energy consumption and use the least amount of power possible during idle. We also observed that the price range of most devices is reasonable and within reach, even for research groups with limited resources. Surprisingly, our search did not identify any commercial smart ring capable of providing raw data useful for research in personalized stress detection. We recommend that researchers consider not only commercial wearables but also explore the latest developments in non-commercial sensors documented in the academic literature, for example, ref. [130] developed OmniRing, an open-source smart ring platform equipped with an Inertial Measurement Unit (IMU) and PPG sensors for activity tracking and health analytics applications. Innovations also include novel sensors specifically designed to measure physiological stress markers, such as cortisol levels throughout the day [131,132]. Recent progress has led to the creation of an electronic skin for stress response assessment [133] that non-invasively monitors three vital signs—pulse waveform, galvanic skin response, and skin temperature—and six molecular biomarkers in human sweat (glucose, lactate, uric acid, sodium ions, potassium ions, and ammonium) for enhanced monitoring accuracy. These sensors offer a promising avenue for capturing more accurate stress responses in naturalistic settings. Often developed in academic or open-source environments, such devices provide unique opportunities for customization and experimentation, extending beyond the capabilities of standard commercial products.

### 4.5. Real-World Challenges

Our examination of the practical challenges associated with stress detection solutions revealed some relevant recurrent themes: data quality, technical limitations, user experience, privacy and transparency. Above all, a key concern pertains to the quality and inherent distortions present in raw data obtained from wearable devices. Notably, signal distortions caused by motion artifacts, physical or human activity, and limitations in optical sensors present significant hurdles [113,114,116,118]. While integrating accelerometer data and utilizing human activity recognition classifiers show promise as solutions, tackling optical sensor challenges, particularly with PPG and SKT sensors, proves to be a more complex task which needs further investigation, in particular in the area of distortions given by different reflection patterns of skin (e.g., tattoos, skin tone). Shifting to technical considerations, wearable devices introduce limitations that substantially impact the real-world implementation of stress detection models. Issues such as battery life, computing and network capabilities, and the ongoing challenge of vendor updates necessitate attention. The interplay between information granularity and battery life directly influences data completeness and user experience [111,116]. Recognizing the inherent limitations of wearable devices, the common strategy involves relying on edge layers and cloud solutions to manage complex operations like inference [116]. On the other hand, vendors should grant researchers greater control over the source code of devices, potentially through the adoption of open-source solutions. Moving to user experience and behavioral aspects, the importance of technical support and careful consideration of user data access take center stage. Establishing effective communication channels with study participants becomes crucial, with user-friendly wearables helping to mitigate technical issues and enhancing the overall user experience [114,121]. During the data labeling stage researchers should also pay more attention to optimizing the balance between accuracy and the burden on study subjects, possibly by introducing machine learning models for identifying the ideal timing for the user to send the request for the label [54,121]. Simultaneously, the commitment to privacy by design principles emerges as a foundational pillar. This commitment involves securing informed participant consent, restricting data collection to essential information, and excluding non-essential data. In this context, proposals for on-device stress detection models surface as potential privacy-mitigating strategies, although their feasibility necessitates careful consideration of current technological constraints. The medical context of stress detection also necessitates transparent models that describe decision-making processes. White box models, particularly those employing SHAP values, gain favor for their transparency. However, they are limited to classical machine learning approaches, leaving a noticeable gap in interpretable models for deep learning in personalized stress detection. This emphasizes the need for sustained research in this specific area.To sum up, the diverse challenges uncovered in our exploration call for a comprehensive and collaborative strategy. Ongoing research, interdisciplinary collaboration, and commitment to ethical and transparent practices are crucial in overcoming these challenges and nurturing the development of effective and responsible stress detection models applicable in the real world.

## 5. Conclusions

In conclusion, this paper provides a comprehensive overview of the evolving landscape of personalized stress detection models, focusing on using wearable devices to undertake the task. The synthesis of findings in this scoping review addressed the current literature gap and revealed the relationship between biosignals, artificial intelligence methodologies, datasets, wearables, and real-world implementation challenges. The systematic approach employed in this review, guided by the PRISMA-ScR framework, ensures a rigorous examination of the existing knowledge base. As the field of personalized stress detection through wearable technology continues to progress, this review serves as a valuable resource, offering insights into the current state of research, highlighting limitations, and suggesting promising avenues for future exploration. Integrating personalized models into stress detection systems marks a significant advancement, promising tailored interventions that can positively impact individual well-being in various settings, including clinical practice.

## Figures and Tables

**Table 1:** Research questions and related search queries.

Research Question	Queries
What are the primary biosignals provided by wearables that can be utilized for personalized stress detection?	(“personalized” OR “subject level” OR “individual”) AND (“stress”) AND (“detection” OR “prediction” OR “recognition” OR “classification”) AND (“wearable”)
What are the key artificial intelligence techniques used for developing models for personalized stress detection?	(“physiological”) AND (“stress”) AND (“detection” OR “prediction” OR “recognition” OR “classification”) AND (“wearable”)
Are there publicly available datasets fortraining personalized stress detection models?	(“personalized” OR “subject level” OR “individual”) AND (“stress”) AND (“detection” OR “prediction” OR “recognition” OR “classification”) AND (“dataset”)
	(“emotion” OR “stress”) AND (“detection” OR “recognition” OR “research” OR “classification” OR “prediction”) AND (“dataset” OR “database”)
What are the wearable devices available on the market that allow the acquisition of raw data?	Web search: {biosignal} AND “wearable device”
What are the primary challenges encountered in the practical implementation of stress detection modelsin the real world?	(“wearables” OR “wearable devices”) AND (“machine learning” OR “artificial intelligence” OR “monitoring”) AND (“challenge” OR “challenges” OR “issues” OR “perspectives” OR “limitations” OR “topics”) AND (“ethical” OR “ethics” OR “sensors”) AND (“arousal” OR “distress” OR “stress” OR “physiological activity” OR “physiological reactions” OR “physiological response”)
	(“wearables” OR “wearable devices” OR “smartwatch” OR “smartwatches”) AND (“machine learning” OR “ml” OR “artificial intelligence” OR “ai” OR “monitoring” OR “prediction” OR “classification” OR “detection”) AND (“challenge” OR “challenges” OR “issues” OR “perspectives” OR “limitations” OR “topics”) AND (“ethical” OR “ethics” OR “sensors”) AND (“arousal” OR “distress” OR “eustress” OR “stress” OR “physiological activity” OR “physiological reactions” OR “physiological response”)

**Table 2:** Studies about biosignals and techniques for personalized stress detection. Reported performance is the average across all subjects involved in the study.

Study	Year	Sample (Gender)	Type	Signals	Stressor	Task Type	Ground Truth	Approach (Model)	Performance (Metric)	Dataset
[19]	2010	22 (N/A)	SCE	ECG EDA SKT RSP	Public Speaking Stressor Mental Arithmetic Stressors Cold Pressor Stressor	Classification: Binary	EMAs	ML (RBF SVM)	68% (Precision)	N/A
[20]	2015	5 (N/A)	SCE	PPG EDA	Trier Social Stress Test	Classification: Binary	STAI	ML (SVM)	78.98% (Accuracy)	N/A
[21]	2015	8 (N/A)	LIFE	PPG ACC	Real Life	Classification: Binary	EMAs	ML (REPTree)	85.7% (Accuracy)	N/A
[22]	2015	44 (44M, 0F)	LAB	PPG ECG EDA EEG EMG	Go/No-go Visual Reaction Stroop Color Test Fast Counting PASAT Speed Run Visual Forward Digit Span N-back	Classification: Binary	STAI	ML (K-Means + GRNN)	85.2% (Accuracy)	N/A
[23]	2016	6 (N/A)	LIFE	EDA	Real Life	Classification: Binary	Clinical Notes	STAT (Threshold-based Classifier)	60.55% (Accuracy)	N/A
[24]	2017	18 (N/A)	LAB	PPG EDA MIC ACC	Trier Social Stress Test	Classification: Binary	STAI	ML (AdaBoost)	94% (Accuracy)	N/A
[25]	2017	33 (25M, 8F)	LAB	PPG ECG EDA RSP NIBP	Memory Game Fly Sound Image Stimuli Cold Pressor Stressor	Classification: Binary	Experimental Condition	ML (kNN)	95.8% (Accuracy)	N/A
[26]	2018	40 (N/A)	SCE	ECG EDA EMG	Driving Mathematical Questions Analytical Questions	Classification: Binary	Experimental Condition	DL (2*TCN [Shared] + 1 TCN [Subject])	0.918 (AUROC)	[27]
[28]	2019	21 (18M, 3F)	SCE	PPG EDA ACC	Contest	Classification: 3-level	NASA-TLX Free Stress Scale (0–100)	ML( RF/MLP)	97.92% (Accuracy EMP) 91.54% (Accuracy SAM)	N/A
[29]	2020	32 (22M, 10F)	SCE	PPG EDA SKT ACC	Exam	Classification: Binary 4-level	NASA-TLX Experimental Condition	ML (RF)	Binary: 92.5% (Accuracy) 4-level: 85.63% (Accuracy)	N/A
[30]	2020	15 12M, 3F)	LAB	PPG EDA SKT	Trier Social Stress Test	Classification: 4-level	PANAS STAI SAM SSSQ	ML (RF)	96.68% (Accuracy)	[31]
[32]	2020	73 (28M, 45F)	LIFE	PPG ACC	Real Life	Classification: Binary	EMAs	ML (SOM)	54.5% (Accuracy)	N/A
[33]	2020	255 (N/A)	LIFE	EDA SKT ACC	Real Life	Regression: Stress level	EMAs	DL (LC + LSTM DAE)	16.5 (MAE)	[34]
[35]	2020	255 (N/A)	LIFE	EDA SKT ACC	Real Life	Regression: Stress level	EMAs	DL (LC + LSTM DAE)	15.0 (MAE)	[34]
[36]	2021	15 (8M, 7F)	LIFE	ECG	Real Life	Classification: Binary	EMAs	ML (SOM + Fuzzy Classifier)	95.7% (Precision)	N/A
[37]	2021	34 (11M, 23F)	LAB	ECG EMG	Stroop Color–Word Test Math Test	Classification: 3-level	STAI Free Stress Scale (1–5)	ML (Fuzzy Clustering Membership-based Classifier)	75.6% (Accuracy)	N/A
[38]	2021	14 (N/A)	LIFE	PPG ACC GYR	Real Life	Classification: Binary	EMAs	ML (RF)	76% (F1 score)	N/A
[39]	2021	41 (5M, 36F)	LIFE	ECG EDA	Real Life	Classification: Binary	EMAs	DL (MFN with SABs)	77.4% (F1 score)	N/A
[40]	2021	15 (12M, 3F)	LAB	PPG	Trier Social Stress Test	Classification: Binary 3-level	PANAS STAI SAM SSSQ	DL (1D-CNN)	Binary: 82.2% (F1 score) 3-level: 70.5% (F1 score)	[31]
[41]	2022	15 (12M, 3F)	LAB	PPG EDA SKT ACC	Trier Social Stress Test	Classification: Binary	PANAS STAI SAM SSSQ	ML (LR)	99.98% (Accuracy)	[31]
[42]	2022	N/A (N/A)	LIFE	PPG ACC	Real Life	Classification: Binary	EMAs	ML (N/A)	N/A	N/A
[43]	2022	35 (10M, 25F)	LAB	PPG	Stroop Color Test Trier Social Stress Test Hyperventilation	Classification: Binary	STAI PSS	STAT (Adaptive Reference Range-based Classifier)	68.63% (Accuracy)	[43]
[44]	2022	14 (N/A)	LIFE	PPG	Real Life	Classification: 4-level	EMAs	DL (LSTM)	64.5% (Accuracy)	N/A
[45]	2022	15 (12M, 3F)	LAB	EDA	Trier Social Stress Test	Classification: Binary 3-level	PANAS STAI SAM SSSQ	DL (2*1D-CNN + FCN)	Binary: 90% (Accuracy) 3-level: 70% (Accuracy)	[31]
[46]	2022	15 (12M, 3F)	LAB	PPG	Trier Social Stress Test	Classification: Binary 5-level	PANAS STAI SAM SSSQ	DL (1D-CNN)	Binary: 96.7% (Accuracy) 5-level: 80.6% (Accuracy)	[31]
[47]	2023	15 (0M, 15F)	LIFE	PPG EDA SKT ACC	Real Life	Classification: Binary	EMAs	DL (CNN Architecture with SSL)	79.65% (Accuracy)	[48]
[49]	2023	41 (34M, 7F)	LAB	ECG EDA RSP NIBP	Fire Response Task in VR N-back	Classification: Binary	Free Stress Scale (0–100)	ML (RF)	82% (Accuracy VR) 98% (Accuracy N-back)	N/A
[50] *	2023	15 (12M, 3F)	LAB	EDA	Trier Social Stress Test	Regression: Items in scales	PANAS STAI SAM SSSQ	DL (CNN Architecture with SSL)	N/A	[31]
[51] *	2023	15 (12M, 3F)	LAB	ECG EDA EMG SKT RSP ACC	Trier Social Stress Test	Classification: 3-level	PANAS STAI SAM SSSQ	DL (1D CNN + MLP)	95.06% (Accuracy)	[31]
[52]	2023	16 (8M, 8F)	LAB	EDA SKT	Acoustic Stressors	Classification: Binary	Experimental Condition	STAT (MOS Algorithm [53])	92.74% (Accuracy)	N/A
[54]	2023	20 (13M, 7F)	LIFE	PPG ACC GYR	Real Life	Classification: Binary	EMAs	ML (RF)	40–45% (Recall)	N/A
[55]	2023	83 (51M, 32F)	LIFE	PPG EDA SKT ACC	Real Life	Classification: Binary	EMAs	ML (RF)	66.55% (Accuracy)	N/A

* Preprint.

**Table 3:** Biosignals, connection to stress, and number of studies employing each biosignal.

Signal	Description	Connection to Stress	N
EDA	EDA is a biosignal that measures skin conductance, reflecting sweat gland activity.	Activation of the sympathetic nervous system during stress stimulates eccrine sweat glands through the release of neurotransmitters (especially norepinephrine) [63], leading to sweat production and changes in skin conductance.	21
PPG	PPG is a biosignal that records changes in the volume of blood flow in arteries, capillaries, and any other tissue following each contraction and relaxation of the heart.	Stress hormones such as cortisol and adrenaline, released during stressful situations, activate the sympathetic nervous system (SNS). This activation results in an increased heart rate, stronger cardiac contractions, and vasoconstriction, especially in the extremities [64], affecting blood flow and PPG readings.	19
SKT	SKT is a biosignal that measures the temperature of the skin in the peripheral area.	Skin temperature is closely linked to blood flow and it is affected by peripheral vasoconstriction induced by stress hormones like cortisol and adrenaline [65]. In acute stress, a slight decrease in temperature, attributed to vasoconstriction, is expected [66].	10
ECG	ECG is a biosignal that records the electrical activity of the heart on the surface of the body during the cardiac cycle.	Sympathetic nervous system activity primarily involves an increase in heart rate during stress [67]. ECG, like PPG, provides insights into these changes, including alterations in PR interval and QRS duration [68].	9
EMG	EMG is a biosignal that records the electrical activity produced by the muscle when it contracts.	Cortisol and adrenaline, released during stress, induce a phenomenon of muscular vasodilation, resulting in muscle tension that prepares the body for action or damage minimization [69].	4
RSP	RSP is a biosignal that provides information about the patterns of inhalation and exhalation in an individual.	During stressful situations, as a component of the fight-or-flight response, the body readies itself for either escape or confrontation by dilating the airways and altering respiration patterns. These responses, as seen for other biosignals, are directed by the influence of the HPA axis [70].	3
NIBP	NIBP is a biosignal that measures blood pressure without the requirement for invasive procedures.	Stress impacts blood pressure patterns through the activation of the HPA axis, leading to pressure fluctuations resulting from increased heart rate and blood vessel constriction [71].	2
EEG	EEG is a biosignal that measures the electrical activity of the brain, specifically detecting fluctuations in voltage resulting from ionic current flows within the neurons of the brain [72].	Under stress conditions, the brain focuses concentration and increases alertness to enhance the body’s chances of surviving the situation [73,74]. These processes are reflected in an increase in β activity and a decrease in α activity.	1

**Table 4:** Summary of filters used for cleaning, grouped by biosignal.

Filter	Definition	Signal	Studies
Lowpass	A lowpass filter is a filter which allows signals with frequencies below a specified cutoff frequency to pass through, attenuating higher frequencies.	EDA ECG SKT	[23,24,33,35,49,52] [36] [52]
Highpass	A highpass filter is a filter which permits signals with frequencies above a defined cutoff frequency to pass through while attenuating lower frequencies.	ECG EMG	[25] [37]
Bandpass	A bandpass filter is a filter which selectively allows a specific range or “band” of frequencies to pass through, attenuating frequencies outside that range.	PPG ECG EDA EMG NIBP	[24,38,46] [37] [55] [22] [49]
Notch	A notch filter is a filter which attenuates a specific narrow range of frequencies, effectively creating a “notch” in the frequency response.	ECG	[36,49]

**Table 5:** List of machine learning algorithms employed in personalized stress detection.

Algorithm	Tree-Based	Unsupervised + Supervised	N
Random Forest	✓	✗	7
Self-Organizing Map-based Classifier	✗	✓	3
Support Vector Machine	✗	✗	2
AdaBoost	✓	✗	1
Bagging (REPTree)	✓	✗	1
K-Nearest Neighbors	✗	✗	1
K-means + GRNN	✗	✓	1
Logistic Regression	✗	✗	1
Multilayer Perceptron	✗	✗	1

**Table 6:** Common features for each signal with definitions and connection to stress.

Signal	Feature	Definition and Connection to Stress	Studies
PPG ECG	HR	Avg. heart beats per minute; reflects the physiological stress response. Changes in heart rate may indicate the body’s adaptive response to stressors.	[19,21,22,32,37,38,44,54,55]
	RR	Mean duration between R-peaks; reflects the autonomic nervous system interplay. Variations in RR intervals may signify the dynamic balance between sympathetic and parasympathetic branches.	[21,28,29,32,36,37,38,42,54]
	SDNN	Standard deviation of NN intervals; signifies the balance between sympathetic and parasympathetic influences. Changes in SDNN may indicate alterations in autonomic balance and responsiveness to stress.	[21,28,29,36,37,38,54]
	SDSD	Standard deviation of differences in NN intervals; indicates autonomic balance and responsiveness. Variations in SDSD may reflect the regulatory influence of both sympathetic and parasympathetic branches.	[21,28,29,37,38,54]
	RMSSD	Square root of mean squared differences in NN intervals; reflects parasympathetic activity, adaptability, and resilience to stress. Higher RMSSD values are associated with increased adaptability and resilience.	[21,28,29,32,36,37,38,42,49,54]
	pNN50	Percentage of NN intervals differing by >50 ms; indicator of parasympathetic activity and heart regulation. Monitoring changes in pNN50 provides insights into the dynamic regulation of the heart and its response to stressors.	[21,22,28,29,36,37,38,49,54]
	HRV	Variation in time intervals between heartbeats; serves as an indicator of the body’s adaptability to stress. Higher HRV is generally associated with a more flexible autonomic nervous system and better resilience to stressors.	[20,21,22,24,28,29,42,44,49]
	LF	Frequency activity (0.04–0.15 Hz); often associated with sympathetic nervous system activity. LF variations may indicate the sympathetic influence on heart rate during stress.	[19,21,22,28,29,36,37]
	HF	Frequency activity (0.15–0.40 Hz); primarily associated with parasympathetic nervous system activity and respiratory influences. HF variations may indicate changes in relaxation and parasympathetic dominance.	[21,22,28,29,36,37]
	LF/HF	Ratio of low frequency to high frequency; considered a measure of the balance between sympathetic and parasympathetic nervous system activity. A higher ratio may suggest increased sympathetic dominance, potentially indicating stress.	[21,22,28,29,36,37]
EDA	SCL + SCR	Average of combined Skin Conductance Level and Response; comprehensive indicator of arousal. The combined measure reflects both the tonic (baseline) and phasic (event-related) components, providing a holistic view of skin conductance dynamics related to stress.	[20,24]
	SCL	Average Skin Conductance Level (SCL); reflects overall arousal level. SCL provides a baseline measure of sympathetic arousal, contributing to the assessment of stress levels.	[19,25,29,49,55]
	SCL SD	Standard deviation of Skin Conductance Level; indicates variability in arousal. Variations in SCL may suggest fluctuations in the autonomic nervous system’s tonic arousal, possibly linked to stress reactivity.	[19,29]
	SCR	Average Skin Conductance Response (SCR); represents phasic changes in arousal. SCR reflects the rapid, event-related changes in skin conductance, offering insights into acute stress responses.	[22,49]
	SCR Peaks	Number of peaks in Skin Conductance Response; indicates the frequency of arousal events. The count of SCR peaks provides a quantitative measure of how frequently the individual experiences heightened arousal.	[19,29,55]
	SCR Peaks Ampl	Amplitude of peaks in Skin Conductance Response; reflects the intensity or strength of arousal events. The amplitude of SCR peaks may provide information on the magnitude of physiological responses during stress.	[19,25]
RSP	BR	Breathing rate; frequency of breath cycles may indicate stress. Changes in breathing rate can be associated with stress and the body’s effort to adapt to physiological demands.	[25,38,44,54]
	RP	Respiratory period; duration of one respiration cycle may relate to stress. The time taken for a complete respiratory cycle may be influenced by stress-related changes in breathing patterns.	[19,25]
SKT	T	Avg. skin temperature; deviations from the baseline skin temperature may indicate stress. Abnormal skin temperature variations can be indicative of physiological responses to stressors.	[19,55]
	T SD	Standard deviation of skin temperature; variability in skin temperature may be associated with stress. Increased T SD may suggest fluctuations in autonomic responses linked to stress reactivity.	[19]
	T Slope	Slope of skin temperature trends; changes in slope may reflect stress-related temperature dynamics. The rate of change in skin temperature may provide insights into adaptive responses to stressors.	[55]
NIBP	SBP	Systolic blood pressure; elevated SBP may indicate increased stress or heightened physiological response. Systolic blood pressure is sensitive to acute stressors and reflects the force exerted on arterial walls during heartbeats.	[25,49]
	DBP	Diastolic blood pressure; elevated DBP may suggest sustained stress or tension in the cardiovascular system. Diastolic blood pressure reflects the pressure in the arteries when the heart is at rest, and chronic stress may contribute to sustained elevation.	[25,49]
EMG	EMG	Avg. value of muscle activity (EMG); increased activity may indicate stress. EMG captures muscle activity, and elevated average values may be associated with heightened muscle tension or stress responses.	[22,37]
	EMG SD	Standard deviation of muscle activity (EMG); variability in muscle activity may be associated with stress. Increased EMG SD suggests fluctuations in muscle tension, potentially reflecting stress-related changes in motor activity.	[22,37]
EEG	Mean α, β, σ, θ	Mean values of different EEG frequency bands (α, β, σ, θ); variations in EEG frequencies, such as increased beta and decreased alpha, may be associated with heightened mental activity or stress. Changes in delta and theta frequencies could also indicate alterations in relaxation or arousal states.	[22]

**Table 7:** Summary of public datasets for personalized stress detection model development.

Dataset	Type	Sample (Gender)	Biosignals (Device)	Stressor	Ground Truth	Pros and Cons
SWELL-KW [82]	SCE	Size: 25 (17M, 8F) Age: 25 (3.25)	ECG (Movi) EDA (Movi) FBT (Kinect) VS (Camera)	Email interruptions Time pressure	NASA-TLX RSME SAM Free Stress Scale ICI	+ The stress condition mirrors what can be found in real life in a work environment - Potential age and gender bias - It focuses on a specific work-related stress condition
AffectiveROAD [83]	SCE	Size: 10 (5M, 5F) Age: 29.9 (3.7)	PPG (Empatica E4) EDA (Empatica E4) ACC (Empatica E4) ECG (BioHarness 3.0) SKT (BioHarness 3.0) RSP (BioHarness 3.0) VS (Camera)	Real life (Driving)	External annotation: Stress Metric (Observer) Self assessment: Label validation	+ The presence of video streams enables the development of sensorless models (with rPPG) - Very limited sample size - Potential age bias - It focuses on driving stress - The stress metric is only validated by the driver
WESAD [31]	LAB	Size: 15 (12M, 3F) Age: 27.5 (2.4)	ECG (RespiBAN) EDA (RespiBAN) EMG (RespiBAN) SKT (RespiBAN) RSP (RespiBAN) ACC (RespiBAN) PPG (Empatica E4) EDA (Empatica E4) SKT (Empatica E4) ACC (Empatica E4)	Trier Social Stress Test	PANAS STAI SAM SSSQ	+ Solid protocol and assessment of the participants’ state + It includes a wide range of signals from sensors placed both on the chest and the wrist - Potential gender and age bias - The Trier Social Stress Test elicits an extreme response that may not be comparable to those experienced by a non-clinical subject in real-life
CLAS [84]	LAB	Size: 62 (45M, 17F) Age: Mostly 20–27	PPG (Shimmer 3 GSR+) ECG (Shimmer3 ECG) EDA (Shimmer 3 GSR+) ACC (Shimmer 3 GSR+)	Video stimuli Math problems Logic problems Stroop Test	Stimuli label Task performance	+ Fairly big sample size - Potential age and gender bias - No clear details on the age distribution are provided - Researchers did not implement a solid strategy for ground truth
PASS [85]	LAB	Size: 48 (N/A) Age: N/A	ECG (BioHarness 3.0) RSP (BioHarness 3.0) PPG (Empatica E4) EDA (Empatica E4) SKT (Empatica E4) EEG (Muse Headband)	Gaming Physical activity (Cycling)	BORG NASA-TLX (Variant)	+ Combining mental stress and physical activity enables the development of models that account for movement artifacts and discriminate between physical and mental stress - Very limited information about the sample - ACC data not included in the dataset
UBFC-Phys [86]	LAB	Size: 56 (10M, 46F) Age: 21.8 (3.11)	PPG (Empatica E4) EDA (Empatica E4) VS (Camera)	Trier Social Stress Test (Variant)	CSAI	+ The presence of video streams enables the development of sensorless models (with rPPG) - Potential gender and age bias - The Trier Social Stress Test elicits an extreme response that may not be comparable to those experienced by a non-clinical subject in real life
MDPSD [87]	LAB	Size: 120 (72M, 48F) Age: 22 (N/A)	PPG (N/A) EDA (N/A)	Stroop Test Rotation Letter Test Kraepelin Test	Free Stress Scale	+ Fairly large sample size- Potential age bias - Limited information regarding the devices used for data collection - All the stressors elicit an extreme response that may not be comparable to those experienced by a non-clinical subject in real life
SMILE [88] **	LIFE	Size: 45 (6M, 39F) Age: 24.5 (3.0)	EDA (IMEC Chill Band) ACC (IMEC Chill Band) ECG (IMEC Health Patch) ACC (IMEC Health Patch)	Real life	EMAs	+ Real-life stress assessment using EMAs - Potential gender and age bias
EmpathicSchool [89] *	LAB	Size: 20 (N/A) Age: 25.3 (4.3)	PPG (Empatica E4) EDA (Empatica E4) SKT (Empatica E4) IBI (Empatica E4) ACC (Empatica E4) VS (Camera)	IQ test Presentation Stroop Color–Word Test	NASA-TLX	+ It contains a combination of stressors, encompassing both extreme conditions and real-life challenges - Relying solely on NASA-TLX as a ground-truth measure may not provide a reliable identification of stress states
A multimodal sensor dataset for continuous stress detection of nurses in a hospital [48]	LIFE	Size: 15 (0M, 15F) Age: 30–55 (range)	PPG (Empatica E4) EDA (Empatica E4) SKT (Empatica E4) IBI (Empatica E4) ACC (Empatica E4)	Real life (working at the hospital during COVID-19)	Automated labeling using an algorithm trained on AffectiveROAD Post-shift survey for label confirmation, addition, and correction	+ Intelligent automated pre-labeling approach using a pre-trained model + Researchers also investigated the factors causing stress - Very strong gender bias - Potential recall bias - The data pertain to a specific context (a hospital during a pandemic), which may differ significantly from real-life situations
MMSD [80]	LAB	Size: 74 (36M, 38F) Age M: 35 (13) Age F: 33 (12.5)	PPG (Shimmer) ECG (Shimmer) EDA (Shimmer) EMG (Shimmer) GYR (Shimmer)	Stroop Color–Word Test Mental Arithmetic Test Computer Work	STAI Cortisol Test	+ Good sample size+ Sample carefully controlled to be representative of the French population + Ground truth using both a validated scale and an objective gold standard technique (cortisol sample) - All the stressors elicit an extreme response that may not be comparable to those experienced by a non-clinical subject in real life
Stress-Predict Dataset [43]	LAB	Size: 35 (10M, 25F) Age: 32 (8.2)	PPG (Empatica E4)	Stroop Color Test Trier Social Stress Test Hyperventilation	STAI PSS	+ Two validated scales for stress assessment provide a solid label - Potential gender bias - All the stressors elicit an extreme response that may not be comparable to those experienced by a non-clinical subject in real life
AKTIVES [81]	LAB	Size: 25 (10M, 15F) Age: 10.2 (1.27) Clinical sample	PPG (Empatica E4) EDA (Empatica E4) SKT (Empatica E4) VS (Camera)	Gaming	External annotation: 3 observers	+ Using 3 independent annotators mitigates the risk of mislabeling + Clinical sample and control group - Age-specific dataset - External annotation might not be accurate

* Preprint. ** Only available online (no paper nor preprint found).

**Table 8:** Analysis of stressors employed in dataset construction.

Type of Stressor	Stressor	Dataset(s)
Daily life	Gaming Computer work Real life Driving Video/image stimuli Email interruptions Time pressure Public speaking	[81,85] [80,89] [48,88] [83] [84] [82] [82] [89]
Artificial	Stroop Test Trier Social Stress Test Mental Arithmetic Test IQ Test (Variant) Kraepelin Test Rotation Letter Test Hyperventilation Provocation Test	[43,80,84,87,89] [31,43,86] [80,84] [84,89] [87] [87] [43]

**Table 9:** List of devices for raw signal acquisition.

Device	Type	Sensors	Connectivity	Mobile	Release	Battery Life	Cost (Q4 2023)
Empatica EmbracePlus [95]	Wrist	PPG, EDA, SKT, ACC, GYR	Bluetooth	Android, iOS	2020	7 days	∼2000 EUR
Samsung Galaxy Watch 4 [96]	Wrist	PPG, BIA, ACC, GYR	WiFi, Bluetooth	Android	2021	40 h	∼140 EUR
Samsung Galaxy Watch 5 [97]	Wrist	PPG, BIA, ACC, GYR	WiFi, Bluetooth	Android	2022	50 h	∼200 EUR
Samsung Galaxy Watch 6 [98]	Wrist	PPG, BIA, SKT, ACC, GYR	WiFi, Bluetooth	Android	2023	40 h	∼300 EUR
Polar OH1+ [99]	Arm	PPG, ACC	Bluetooth	Android, iOS	2019	12 h	∼60 EUR
Polar H10 [100]	Chest	ECG, ACC	Bluetooth	Android, iOS	2017	400 h	∼90 EUR
Polar Verity Sense [101]	Arm	PPG, ACC, GYR	Bluetooth	Android, iOS	2021	20 h	∼100 EUR
Bangle.js 2 [102]	Wrist	PPG, ACC	Bluetooth	Android	2021	4 days	∼90 EUR
Shimmer3 ExG [103]	Multi	ECG, EMG, ACC, GYR	Bluetooth	Android	2018	N/A	∼550 EUR
Shimmer3 GSR+ [104]	Wrist	EDA, ACC, GYR	Bluetooth	Android	2018	N/A	∼520 EUR
PLUX cardioBAN [105]	Chest	ECG, ACC	Bluetooth	Android	2022	N/A	∼500 EUR
PLUX muscleBAN [106]	Arm	EMG, ACC	Bluetooth	Android	2022	N/A	∼320 EUR
EmotiBit [107]	Arm	PPG, EDA, SKT, ACC, GYR	WiFi, Bluetooth	Android	2022	4-8 h	∼230 EUR
BrainBit Callibri [108]	Multi	ECG, EMG, EEG, ACC	Bluetooth	Android, iOS	2019	24 h	∼280 EUR
BrainBit Headband [109]	Head	EEG	Bluetooth	Android, iOS	2019	12 h	∼460 EUR
Interaxon Muse S (Gen 2) [110]	Head	PPG, EEG	Bluetooth	Android, iOS	2022	10 h	∼310 EUR

**Table 10:** List of studies on real-world challenges in stress detection solutions, grouped by theme.

Theme	Study(s)
Data quality and signal distortion	[111,112,113,114,115,116,117,118,119]
Technical aspects related to wearable devices	[111,116,120]
User experience and behavior	[114,116,120,121]
Privacy	[120]
Interpretability	[118]

## Data Availability

No new data were created or analyzed in this study. Data sharing is not applicable to this article.

## Abbreviations

The following abbreviations are used in this manuscript:

M	Male
F	Female
N/A	Not Available
SD	Standard Deviation
HPA axis	Hypothalamus–Pituitary–Adrenal axis
HRV	Heart Rate Variability
SCE	Controlled Scenario Environment
LAB	Laboratory
LIFE	Real life
ECG	Electrocardiogram
EDA	Electrodermal Activity
RSP	Respiration
SKT	Peripheral Skin Temperature
ACC	Accelerometer
IMU	Inertial Measurement Unit
PPG	Photoplethysmography
EEG	Electroencephalogram
EMG	Electromyogram
SO2	Peripheral Oxygen Saturation
MIC	Microphone
VS	Video Stream
FBT	Full Body Tracking
GYR	Gyroscope
IBI	Interbeat Interval
BIA	Bioelectric Impedance Analysis
NIBP	Non-Invasive Blood Pressure
EMA	Ecological Momentary Assessment
TSST	Trier Social Stress Test
STAI	State Trait Anxiety Inventory
PANAS	Positive and Negative Affect Schedule
SAM	Self-Assessment Manikin
SSQ	Short Stress State Questionnaire
PSS	Perceived Stress Scale
RSME	Rating Scale Mental Effort
ICI	Internal Control Index
CSAI	Competitive State Anxiety Inventory
NASA-TLX	NASA Task Load Index
ML	Machine Learning
DL	Deep Learning
GRNN	General Regression Neural Network
DAE	Denoising Auto Encoder
SVM	Support Vector Machine
RBF	Radial Basis Function
MLP	Multilayer Perceptron
RF	Random Forest
SOM	Self-Organizing Map
AUROC	Area Under the Receiver Operating Characteristic
TCN	Temporal Convolutional Network
LR	Logistic Regression
CNN	Convolutional Neural Network
LSTM	Long Short-Term Memory
SSL	Self-Supervised Learning
MFN	Modality Fusion Network
SAB	Self-attention Block
SHAP	SHapley Additive exPlanations
